# Bringing the Hospital to the Adult Patient: A Qualitative Study of Patients' and Relatives' Experiences of Mobile Emergency Care at Home

**DOI:** 10.1111/scs.70288

**Published:** 2026-06-21

**Authors:** Åsa Falchenberg, Ulf Andersson, Anders Sterner, Gabriella Norberg Boysen, Henrik Andersson

**Affiliations:** ^1^ PreHospen: Centre for Prehospital Research University of Borås Borås Sweden; ^2^ Faculty of Caring Science, Work Life and Social Welfare University of Borås Borås Sweden; ^3^ Academy for Police Work University of Borås Borås Sweden; ^4^ Faculty of Health and Life Sciences Linnaeus University Växjö Sweden

**Keywords:** emergency care, home‐based emergency care, Mobile Emergency Team, Mobile Health Units, patient experience, qualitative research

## Abstract

**Aim:**

To explore how patients and their relatives perceive and experience emergency care delivered at home by Mobile Emergency Teams (METs).

**Design:**

An exploratory qualitative design was used to explore how patients and relatives experienced the care encounter. This approach was appropriate given the exploratory nature of the study.

**Ethical Considerations:**

The study was approved by the Swedish Ethical Review Authority (No: 2023–02186‐01) and conducted in accordance with the Declaration of Helsinki. Participants provided informed consent, were assured of confidentiality, and were informed of their right to withdraw at any time.

**Methods:**

The study was conducted in southwestern Sweden and included 20 semi‐structured interviews with patients (*n* = 11), relatives (*n* = 4), and joint patient‐relative interviews (*n* = 5) who had recently received care from METs. Interviews were audio‐recorded and transcribed verbatim. Data were analyzed using Braun and Clarke's six‐phase thematic analysis.

**Results:**

The analysis generated two overarching themes: *Embraced by a calming environment* and *A sense of safety and security.* Participants described METs' arrival as a turning point that brought calm and clarity to emotionally intense situations. The teams' respectful approach, clear communication and structured assessments contributed to emotional reassurance. Receiving care at home, in a familiar environment and in the presence of loved ones, supported participants' sense of dignity, autonomy and control. However, some participants expressed uncertainty about what would happen after METs' departure, indicating a need for improved follow‐up and continuity of care.

**Limitations:**

Findings are shaped by a specific Swedish context, joint interviews and purposive sampling, which may limit transferability.

**Conclusion:**

Emergency care at home was experienced as emotionally supportive and clinically competent. Attentiveness, clarity and a respectful presence were central to participants' sense of being thoroughly and considerately cared for.

## 1 Introduction

Healthcare systems are facing increasing pressure due to aging populations and a growing number of individuals living with chronic and complex health conditions [1]. At the same time, emergency departments (EDs) are experiencing rising demand, often resulting in overcrowding, extended wait times and fragmented care experiences [2]. In response, both national and international healthcare systems are exploring alternative models for organizing emergency care [3]. One such approach involves delivering emergency care outside of hospital settings and directly into patients' homes [4].

Various models have been introduced to support this shift including Hospital at Home [5], Same Day Emergency Care [6], Acute Hospital Care at Home [4] and Mobile Emergency Team (MET) model [7]. While these models share the aim of reducing hospital admissions and improving care efficiency, they differ in organisation, clinical scope and context of use within emergency care. Hospital at Home and Acute Hospital Care at Home are primarily admission‐avoiding or admission‐substituting models, delivering hospital‐level care in patients' homes over a defined period for selected conditions. Same Day Emergency Care focuses on rapid assessment and treatment within the hospital, enabling discharge without overnight admission [6]. In contrast, the MET model operates at the interface between prehospital and community‐based emergency care, providing on‐site assessment and treatment directly in the patient's home and supporting real‐time decisions regarding the appropriate level of care, including the need for escalation when home‐based care is not appropriate [7].

This study focuses specifically on the MET model, which is currently emerging in Sweden as a way to provide emergency care in patients' homes. In this model, emergency care is delivered by nurses and physicians to patients experiencing sudden illness or injury [8]. METs operate wholly or partially from hospital‐affiliated emergency departments and provide clinical assessment, treatment and follow‐up in patients' homes. The MET model functions as an extension of hospital‐based emergency care and is activated through established emergency care pathways, such as referrals from ambulance services, primary care, or emergency departments [7, 9].

Providing emergency care in the home is not solely a logistical solution; it reflects a broader reconceptualisation of how and where emergency care is delivered. Instead of transporting patients to the hospital, care is brought to the patient. This approach, often referred to as home‐based emergency care, has gained traction internationally [2] and is increasingly being implemented in Sweden [9]. It is particularly relevant for older adults, who may struggle with sudden health changes due to frailty, cognitive impairment and limited mobility [10].

In contrast to traditional emergency care pathways, such as ambulance services and emergency departments, that have historically been organised around rapid response, transport and high patient throughput, the MET model enables clinical assessment, decision‐making and treatment in patients' home. Ambulance services have traditionally focused on rapid response and transport [11] while emergency departments are often described as crowded and time‐pressured environments, where care is shaped by rapid patient flow and protocol‐driven practices that may limit patients' opportunities for involvement [12].

The MET model represents a different organisational approach to emergency care, where hospital‐based emergency competence is delivered in patients' homes instead of within the emergency department. Rather than focus on transport or triage, METs assess, treat and make clinical decisions on site. This shifts emergency care from the hospital to the patient's everyday environment and changes professional roles, decision‐making, and the care encounter.

From a caring perspective, the home is understood not merely as a physical setting, but as a lived space and relational space that shapes how illness, vulnerability and care are experienced [13]. Concepts such as autonomy, dignity, continuity, and safety are thus not solely individual attributes or outcomes, but relational and context‐dependent dimensions emerging within the care encounter [13]. Autonomy in this sense involves the patient's opportunity to participate meaningfully in decisions and care processes, supported by the healthcare professional's attentiveness, responsiveness and clinical judgment. Similarly, dignity and safety are closely linked to experiences of being acknowledged, respected and taken seriously, particularly in situations of sudden illness and dependence.

Beyond these caring dimensions, home‐based emergency care has also been highlighted as a response to increasing pressures on emergency departments and hospital‐based services. Models such as Hospital at Home, Same Day Emergency Care, Acute Hospital Care at Home and METs have been shown to reduce unnecessary hospital admissions, shorten lengths of stay and improve patient flow, thereby contributing to system‐level efficiency and alleviating emergency department crowding [4, 5, 6]. In this sense, care delivered in the home may generate a dual benefit: support both patients' experiences of autonomy and safety while simultaneously addressing structural challenges within contemporary emergency care systems.

In home‐based emergency care, these dimensions may be enacted through patients' involvement in assessments and decisions, the possibility to influence the pace and focus of care and caring as well as the preservation of everyday routines and relationships. For older adults, receiving emergency care at home may contribute to a sense of continuity and existential coherence, as the familiarity of the home environment can mitigate stress, disorientation and feelings of loss of control often associated with hospital‐based emergency care [14, 15]. Receiving care in this environment may foster emotional safety, personal dignity and existential coherence. For older adults, remaining at home during illness can help preserve daily routines, maintain social bonds and reduce the disorientation commonly associated with hospital admissions [16, 17]. In addition to these experiential benefits, home‐based emergency care has been associated with reduced clinical risks, including infections, immobility and hospital‐related complications [18, 19, 20].

Despite these benefits, home‐based emergency care also presents challenges. Most homes are not designed for medical interventions and may require adaptation. The presence of medical personnel and equipment can temporarily disrupt the patient's sense of privacy and control [21]. Moreover, the home environment may lack diagnostic tools, specialist access and immediate resources typically available in hospital settings [22]. However, these limitations may be counterbalanced by a more personalised experience, which can reduce stress and increase comfort [20]. These conditions place demands on how emergency care is delivered in the home, including how professional competence, communication and decision‐making are enacted in relation to patients and their relatives. At the same time, previous research suggests that care provided in the home environment may enable a more personalised care encounter, which can contribute to reduced stress and increased comfort [20].

While the existing literature acknowledges both the potential and limitations of home‐based emergency care, there remains a gap in understanding how patients and their relatives experience this form of care when delivered by METs. Knowledge remains limited regarding how aspects of care, such as safety, autonomy and care quality, are experienced and understood in the context of emergency care provided in the home. Therefore, this study will explore patients' and relatives' experiences of emergency care delivered by METs, with the intention of contributing a deeper understanding of how care is experienced and enacted in this setting.

## 2 Aim

The aim of this study was to explore how patients and their relatives perceive and experience emergency care delivered at home by METs.

## 3 Method

### 3.1 Design

This study used a qualitative, inductive design [23], to explore the perceptions and experiences of patients who received emergency care at home via METs, as well as those of relatives who were present during the care encounter. Semi‐structured interviews were conducted to capture rich, in‐depth insights into how emergency care was experienced in the home setting. Interviews were conducted either individually with patients, jointly with patients and their relatives, or solely with relatives when patients were unable to participate.

### 3.2 Settings and Participants

The study was conducted in southwestern Sweden and involved participants who had received emergency care at home as an alternative to hospital‐based treatment. The MET service targeted adults (≥ 18 years) with suddenly occurring conditions considered suitable for assessment and treatment at home. Care was delivered by two MET organizations affiliated with regional hospitals. These teams, composed of nurses and physicians, operated from hospital EDs or ambulance services and served both urban and rural populations. Their primary task was to assess and treat patients at home who presented new or worsening symptoms and signs of illness or injury. When patients or relatives contacted the national emergency number, they were informed by the dispatcher that a MET could be dispatched to their home, provided the situation was deemed appropriate and consent was obtained.

Participants included patients who had received emergency care from either MET or relatives who were present during the care encounter. Recruitment took place during MET visit, where participants received written information outlining the study's aim and procedures. They were informed that they would be contacted within a few days by ÅF to discuss potential participation. In total, 35 patients or relatives were invited to take part in the study.

Twenty interviews were conducted in total; eleven were individual interviews with patients, four were with only relatives, and five were joint interviews involving both patients and their relatives. Participants were evenly distributed between urban (*n* = 10) and rural (*n* = 10) areas. The patients ranged in age from 55 to 101 years, and the sample included 11 men and nine women (see Table 1). The time between receiving emergency care and the interview ranged from one to nine days, with a median of three days.

**TABLE 1:** Participant characteristics and interview details.

No	Interview participant(s)	Patient's age (years)^a^	Days between care and interview	Patients sex	Interview location	Interview length (min)
1	Wife and daughter	97	5	Male	Home	38
2	Patient and wife	85	8	Male	Home	37
3	Patient and daughter	101	9	Female	Home	48
4	Patient and wife	79	2	Male	Home	33
5	Daughter	85	6	Male	Telephone	28
6	Patient	55	9	Female	Telephone	23
7	Patient	90	2	Male	Telephone	14
8	Patient	81	3	Female	Telephone	26
9	Patient	83	3	Female	Telephone	17
10	Daughter‐in‐law	85	3	Male	Telephone	19
11	Daughter	89	1	Male	Telephone	36
12	Patient	78	1	Female	Telephone	31
13	Patient	91	1	Female	Telephone	11
14	Patient	76	1	Female	Telephone	21
15	Patient	83	1	Male	Telephone	11
16	Patient	77	1	Male	Telephone	11
17	Patient	78	1	Male	Telephone	16
18	Patient	92	3	Female	Telephone	16
19	Wife	88	3	Male	Telephone	22
20	Patient and wife	72	7	Male	Telephone	38

^a^ Age refers to the patient who received care, regardless of whether the interview was conducted with the patient or a relative.

Several individuals declined participation: 11 patients cited poor health, three initially expressed interest but did not attend their scheduled interviews, and one participant withdrew during the interview.

A convenience sampling strategy was used to recruit patients and relatives with direct experience of receiving emergency care at home from MET. Eligible participants were required to be able to take part in an interview and to reflect on their perceptions and experiences of the care encounter. Those who expressed interest were scheduled for an interview at a time and place of their choosing. Informed consent was obtained either in writing or verbally.

### 3.3 Data Collection

The interviews were conducted between September 2023 and January 2024 and were audio recorded. Informed consent was obtained either in writing or verbally, depending on the participant's circumstances.

Each interview began with one open‐ended question: ‘Can you tell me about your experiences of having your care needs met at home by METs?’ This opening allowed participants to narrate their experiences freely. Follow‐up prompts such as ‘Can you tell me more?’ or ‘Can you elaborate on that?’ were used to deepen the exploration of relevant aspects and shaped by participants' responses and guided by the study aim.

In some interviews, relatives participated together with patients. In these interviews, both patients and relatives were invited to share their perceptions and experiences of the emergency care encounter. In cases where patients had difficulties articulating their experiences, for example due to illness‐related fatigue or a reduced capacity to express their perceptions and experiences, relatives contributed by complementing and elaborating on the shared experience of receiving care at home. When interviews were conducted solely with relatives, this occurred because patients were unable to participate due to their health condition.

This study did not distinguish between statements made by patients and those made by relatives, but to capture perceptions and experiences of emergency care at home as a shared and relational care encounter. Relatives' contributions were therefore understood as part of the context in which care was experienced, rather than as separate analytical units. This approach was considered ethically appropriate, as it could support patients' ability to participate in the interview while respecting their autonomy and well‐being.

In total, 20 interviews were conducted, comprising 8 h and 15 min of recorded material with interview lengths ranging from 11 to 48 min and an average duration of approximately 25 min. Variation in interview length reflected participants' health status and capacity at the time of the interview. Four interviews took place in participants' homes, while 16 were conducted via telephone to minimise infection risk (including COVID‐19) and protect older, frail individuals. Participant characteristics and interview conditions are presented in Table 1. Interviews were conducted by ÅF, a registered nurse and PhD student with prior training and experience in qualitative research interviewing. The author group included researchers with extensive experience in emergency care and qualitative methodology.

### 3.4 Data Analysis

Interviews were transcribed verbatim and the analysis followed the six‐phase approach to thematic analysis outlined by Braun and Clarke [24]. The data were analyzed between January and March 2024. Coding and theme development were performed using Microsoft Excel. This method was chosen for its structured yet flexible framework, suitable for exploring patterns of meaning within qualitative data. The analytical process began with repeated readings of the transcripts to gain familiarity with the material, during which initial notes and impressions were recorded. Meaningful segments of text were identified and inductively coded using Excel, allowing for systematic organisation. Codes were then reviewed and clustered into subthemes and main themes, reflecting the participants' experiences of receiving emergency care at home from METs (see Table 2). The development of themes was iterative, involving continuous comparison and refinement to ensure clarity, internal consistency, and distinction between themes.

**TABLE 2:** Examples of data analysis.

Meaning unit	Condensed meaning unit	Code	Subtheme	Main theme
…waiting by the window… waiting for the ambulance with all their equipment, rushing in … this time it didn't feel as dramatic. When MET arrived, it was calmer, and I could pause and process my feelings a bit. I didn't have to think about: Should I get dressed? Should I go with him? Where will he end up? What will happen? I also felt that if they [referring to MET] thought he needed to go to the hospital, they would say so, instead of me becoming hysterical	When the ambulance comes, it usually feels rushed and dramatic, but when MET arrived, it was calmer. I could pause, process my feelings, and trust that if hospital care was needed, they would tell me, instead of me panicking	Calm presence reduced emotional overwhelm	Struggling with the decision to seek help	Embraced by a calming environment
They [referring to MET] took their time to examine carefully, listened to the heart and lungs, took blood samples, and observed him closely	They took their time, examined thoroughly, by listening to his heart and lungs, took blood samples, and observed him closely	Taking time for careful assessment	Experiencing calm and structured care	
That they[referring to MET] came to my home was crucial, especially when you can't see or walk on your own…	That MET coming to my home was crucial, especially when you can't see or walk	Maintaining independence	The importance of remaining in a familiar environment	A sense of safety and security at home
This worry and sense of confusion, wondering if I was doing the right or wrong thing, and how to move forward, I felt I could set aside once I received both verbal and written information about what to do next. That felt very reassuring	The worry and confusion about what to do eased once I received clear verbal and written instructions, which felt very reassuring	Clarity reduced uncertainty	Feeling involved in the care encounter	

Initial coding was conducted by ÅF. The codes and preliminary interpretations were subsequently discussed within the author group to challenge interpretations and support consensus. Throughout the analytical process, the author group was engaged in reflexive discussions to reflect on their preunderstandings, shaped by clinical experience in emergency and home‐based care as well as by caring perspectives. These discussions enhanced credibility, reduced individual bias, and supported a nuanced interpretation of the data [25]. To ensure transparency and comprehensive reporting, the COREQ checklist was used to guide the presentation of the study [26].

### 3.5 Ethical Considerations

The study was conducted in accordance with the ethical principles outlined in the Declaration of Helsinki [27] and was approved by the Swedish Ethical Review Authority in Stockholm (No: 2023‐02186‐01), as well as by relevant clinical operations managers. Participants received written and verbal information about the study, including their right to withdraw at any time without providing a reason. They were assured that participation involved no risks beyond potential emotional discomfort during the interview and they retained full control over what type of information they chose to share.

As participants had recently experienced emergency care in their homes, they could be considered potentially vulnerable. Attention was therefore paid to protect their voluntariness and autonomy throughout the research process. Participants were reminded that participation would not affect their future care, and interviews were conducted with sensitivity to signs of fatigue or distress. Recruitment took place after the MET care encounter had been completed in order to maintain a clear distinction between care and research participation. When relatives participated, the interviewer ensured that each participant was invited to express their own perception and experiences to protect individual autonomy and prevent one voice from overshadowing another. Conducting research in participants' homes required special ethical awareness, as the home represents a private space. Clear boundaries between care and research were maintained, and participants were given control over the time and setting of the interview.

Data handling adhered to the Swedish Data Protection Act [28], and all participant information was anonymised. The code list linking identities to transcripts was stored separately on a password‐protected memory card in a locked cabinet. These measures ensured privacy, confidentiality, and compliance with established ethical research standards [27].

## 4 Results

**FIGURE 1:**
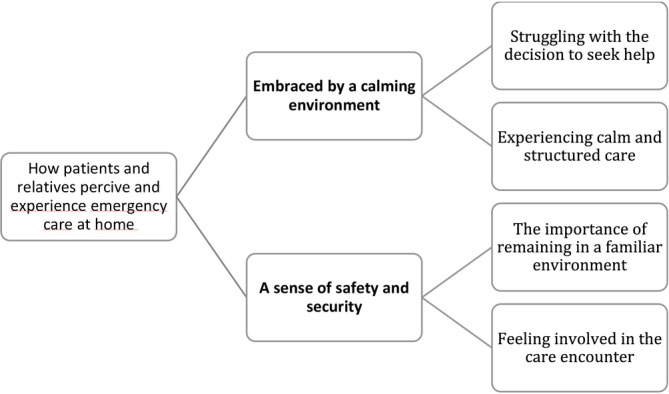
Overview of the main themes and subthemes illustrating patients' and relatives' perceptions and experiences of emergency care at home.

The thematic analysis explored how patients and relatives perceived and experienced emergency care delivered at home by METs. Two main themes were identified: ‘Being embraced by a calming environment’ and ‘A sense of safety and security at home’. Each theme is described through subthemes that reflect both the challenges and the benefits participants associated with home‐based emergency care (see Figure 1).

### 4.1 Embraced by a Calming Environment

This theme illustrates the emotional trajectory from hesitation and uncertainty to relief and reassurance. It captures how METs' presence transformed a situation marked by moral doubt and tension into one characterised by calm, structure, and restored confidence. It is described through two subthemes: ‘Struggling with the decision to seek help’ and ‘Experiencing calm and structured care’.

#### 4.1.1 Struggling With the Decision to Seek Help

Reaching out for help was emotionally difficult and often filled with hesitation. Patients and relatives spoke of internal negotiations, questioning whether symptoms were serious enough or if they were overreacting. Hesitation was often rooted in a reluctance to burden emergency services or to be perceived as exaggerating the situation. Several participants described minimizing their symptoms or waiting in the hope that the condition would improve on its own. The decision was not solely medical but intertwined with moral considerations about responsibility, independence, and not ‘making a fuss.’ One patient reflected: ‘Yes… but first, I want to say that this was the first time I had ever called the national emergency number, … I didn't know… am I sick enough?’ (Interview 19). Help‐seeking was thus described as more than a medical decision; it involved weighing whether the situation justified contacting emergency services.

Relatives often felt torn between honoring the patient's wishes and ensuring their safety. They described monitoring subtle changes over time and wondering whether the situation had crossed a threshold that justified calling for help. This process was emotionally exhausting, particularly when symptoms were ambiguous. One relative explained: ‘I could see something wasn't right, but he kept saying he was fine. I didn't want to go against him, but I was afraid to wait any longer.’ (Interview 5). The possibility of receiving assessment at home made the decision easier for some participants.

When the MET vehicle arrived outside the home, many described an immediate sense of relief. The arrival validated their decision and eased doubts about whether they had overreacted. One relative expressed this moment: ‘When I saw the car park outside, I thought… Oh, thank you!’ (Interview 19).

#### 4.1.2 Experiencing Calm and Structured Care

The arrival of METs marked a clear emotional turning point, transforming the atmosphere from tension and uncertainty to one of calm and reassurance. Participants described a noticeable shift in atmosphere. One participant described: ‘When they came in, everything just settled. It felt like someone finally took control of the situation.’ (Interview 19). METs' calm presence and respectful approach helped de‐escalate anxiety. Their approachability encouraged dialogue and facilitated trust. Even without advanced technology, assessments were perceived as competent and reliable.

Patients and relatives emphasised METs' calm and methodical approach throughout the visit. Examinations were performed step by step and explained using everyday language. As one patient expressed: ‘They told me what they were doing the whole time. Nothing felt rushed or unclear.’ (Interview 6). Clear communication helped participants feel informed, involved, and understood. Participants appreciated how practical and understandable the guidance provided was, what symptoms to monitor, when to seek further care, and how to manage the situation at home. This replaced uncertainty with confidence. This sense of calm made it easier to ask questions and express concerns. The structured approach extended beyond communication to decision‐making. The presence of a physician enabled immediate and confident clinical judgments, relieving relatives from the burden of having to determine whether hospitalisation was necessary. Participants described how decisions were presented clearly and with well‐reasoned explanations, leaving little room for doubt. As one patient reflected: ‘I believe the decision not to go to the hospital was correct…’ (Interview 14).

Participants described how hearing the METs' composed and compassionate communication contributed to relatives' feeling included, even when they were not involved in clinical discussions. One relative recalled: ‘I sat in the kitchen with my mom, and we heard them talk, how sick he really was. But also, they wanted to try, not to give up. That meant everything…’ (Interview 1). This indirect access fostered trust and understanding among both patients and relatives. Immediate feedback on tests helped reduce anxiety. Participants contrasted this with past hospital experiences, which they described as impersonal, noisy and rushed. One relative reflected: ‘At the hospital, everything feels rushed and noisy. My mom has both visual and hearing impairments, and it becomes overwhelming for her. At home, it was calm and they had time to talk.’ (Interview 3). In contrast, being at home was associated with a sense of dignity and calm, grounded in familiar surroundings and the ordinary elements of daily lives. Even when challenges occurred, such as difficulty drawing blood, participants described the team's composure as reassuring and stabilizing. When hospitalisation was necessary, transitions were described as seamless. For those remaining at home, follow‐up contact reinforced a sense of structured and continuous care. However, when follow‐up was inconsistent, uncertainty resurfaced.

### 4.2 A Sense of Safety and Security

This theme is illustrated by two subthemes: ‘The importance of remaining in a familiar environment’ and ‘Feeling involved in the care encounter’. Participants described how care at home preserved dignity and control, promoted inclusion and strengthened emotional resilience. Safety emerged not solely from clinical intervention but from the interaction between place, presence and relational engagement.

#### 4.2.1 The Importance of Remaining in a Familiar Environment

Patients and relatives consistently described the home as a place of emotional calm, control and familiarity during moments of medical uncertainty. Being cared for in their own environment helped reduce stress and made the experience feel more manageable compared to the often chaotic, impersonal and rushed atmosphere of the ED. Safety was thus spatially grounded, rooted in the predictability and personal meaning embedded in the home environment. One relative shared: ‘I didn't have to panic or worry about what would happen. When MET arrived, they came into our home, calmly and reassuringly. They asked if he wanted to stay in his chair or move to the bedroom… It felt like they took care of both him and me…’ (Interview 19).

Avoiding hospital transport was especially important for those with mobility challenges, sensory impairments, or cognitive difficulties. Not having to arrange transport or navigate unfamiliar hospital settings was experienced as relieving. Remaining in their own chair, bed, or living room was described as comforting, supporting orientation and reducing anxiety. Familiar settings like sitting in their own chair or being wrapped in their own blanket, made care feel deeply personal and far less intimidating. One relative noted: ‘If he'd been hospitalised, I wouldn't have known anything. But here at home, I could be near him the whole time. I heard what they said, I could ask questions, and I felt like I was part of it, not just waiting outside somewhere.’ (Interview 2). This highlights how physical proximity in the home setting supported emotional closeness and reinforced a perception of shared care responsibility. Remaining at home appeared to redistribute vulnerability, reducing both practical strain and emotional exposure.

The home setting, with its slower pace and intimate atmosphere, was also perceived as making space for more humane interaction, which participants with previous hospital experiences noted. The contrast with hospital settings further illuminated how safety was associated with tempo, familiarity and relational continuity rather than solely with medical resources. One patient reflected: ‘At home, it felt like they had time for me. I could ask questions, and they explained everything properly, what to look out for, how to take the medicine, and when to get help. It made me feel safer once they left.’ (Interview 9).

Relatives emphasised how remaining at home allowed them to stay present and engaged throughout the care encounter. Physical proximity enabled relational participation, allowing safety to be experienced collectively rather than individually. The familiar setting fostered a sense of continuity and closeness, reinforcing emotional connection. Compared to hospital admissions, where communication could feel fragmented or delayed, participants described feeling better informed and more connected to both the patient and the clinical process. This physical proximity translated into a stronger perception of shared responsibility and support. As one relative expressed: ‘We sat in the living room, my sister and I, and I could hear what the nurse and physician were saying. We were all gathered and I didn't feel left outside. I felt involved the whole time.’ (Interview 1).

For participants with limited social support or increased vulnerability, receiving emergency care at home was particularly significant. Some described feeling exposed or overwhelmed in unfamiliar healthcare environments and emphasised how being met in their own space reduced that sense of fragility. One patient expressed: ‘I am very vulnerable, so it was really good that they could come to my home.’ (Interview 6). In this way, the home functioned as a protective environment that supported emotional stability.

Although participants overwhelmingly preferred to remain at home, they also expressed trust in METs' ability to make appropriate decisions regarding hospitalisation when needed. What mattered most was that these decisions were made on site, together with the team, in a way that felt appropriate and respectful of the situation. For some, remaining at home was not only about comfort but about identity and belonging. Home represented continuity, autonomy, and a deeply personal sense of belonging. One patient expressed: ‘Being at home and enjoying my flowers and patio… I would like to pass away here.’ (Interview 14). This illustrates how safety was intertwined with existential security and the desire to remain in a meaningful environment.

#### 4.2.2 Feeling Involved in the Care Encounter

Participants described how being cared for at home enabled both patients and relatives to feel not only physically present but emotionally involved in the care encounter. This proximity allowed for deeper engagement, clarity, and a shared sense of purpose in navigating the situation together. Relatives expressed that they were not pushed to the margins but were instead invited into the conversation and treated as important collaborators. One shared: ‘They spoke directly to us, not just to my dad. They explained what the symptoms could mean and what the next steps might be. It helped me understand what was going on and why decisions were being made. I felt like I was part of the care, not just watching from the sidelines.’ (Interview 5).

Several participants described how the team addressed both the patient and the relative, ensuring that each had space to speak. One relative noted: ‘They spoke to my husband first, then turned to me with the same calm attentiveness. They let me finish explaining everything, which really helped me focus.’ (Interview 19). This deliberate inclusion reinforced a sense of shared participation rather than hierarchical communication.

Despite the overall positive experience, some participants expressed concern about what would happen after METs left. It was not always clear if follow‐up would occur or what to expect in the coming days. This uncertainty created tension between the calm of the encounter and uncertainty about what lay ahead. One participant reflected: ‘I didn't quite know what the next step would be, whether there would be a referral, a follow‐up call, or if I just had to wait’ (Interview 4). Nevertheless, many felt that the advice and guidance they received helped them regain a sense of control. Instructions on how to monitor symptoms, administer medications, and when to seek further help were appreciated and described as essential to managing aftercare with confidence.

Participants valued being able to describe the situation in their own words without interruption. One patient expressed: ‘They took time to pinpoint the problem, letting us explain everything.’ (Interview 17). Being allowed to narrate the situation strengthened their sense of involvement and ownership in the care process.

Participants associated the home setting with feeling seen and acknowledged, not only as patients or caregivers, but as people. As one patient expressed: ‘They didn't just treat my symptoms. They looked at me, listened, and spoke to me as a person.’ (Interview15). Emotional safety was described as emerging not only from medical competence, but also from being treated with respect, warmth, and attentiveness. In this way, involvement was not merely practical but relationally rooted in a shared moment of presence and care. However, not all participants experienced the same level of inclusion. One patient described moments when clinical discussions occurred above their head: ‘The team discussed among themselves, and it went over my head, but I didn't have the energy to care.’ (Interview 20). These accounts illustrate how involvement could fluctuate depending on patients' physical and cognitive capacity.

## 5 Discussion

The results were presented in two themes: *Embraced by a calming environment* and *A sense of safety and security at home*. Together, these themes illuminate how emergency care delivered at home by METs fostered calm, dignity, and perceived safety through relational presence, contextual sensitivity, and continuity. Safety appeared to be constructed during the encounter, indicating that it may be relationally and contextually produced rather than solely dependent on institutional settings. This discussion explores how these elements contributed to emotional reassurance, perceived stability and overall quality of care as described by patients and relatives.

In the theme *Embraced by a calming environment*, METs' arrival marked a turning point, transforming a previously overwhelming situation into one characterised by calm and clarity. This shift in atmosphere was not solely attributable to clinical expertise but to how that expertise was enacted through the interpersonal presence of the team. Respectful interaction, tone of voice and structured communication did not merely accompany treatment; they actively shaped how the situation was perceived and experienced by patients and relatives. Calmness emerged as a relational achievement rather than a passive emotional response that transformed the experience, making patients and relatives feel seen and supported.

Participants emphasised that clear, respectful communication was essential to emotional reassurance and overall confidence in the care encounter. Explanations of clinical decisions and attentive responses to questions significantly shaped how care was perceived. Previous research similarly highlights that relational communication enhances emotional safety and confidence, particularly in high‐pressure emergency settings [29]. In this study, a calm tone, respectful demeanor and personalised interaction often appeared as central as the clinical intervention itself. Communication should therefore be understood not as an adjunct to emergency care, but as constitutive of how safety and professional competence are experienced. By stabilizing uncertainty and providing orientation in moments of crisis, relational communication functioned as an anchor within a vulnerable situation and reinforced the feeling of being genuinely cared for. This relational dimension was not merely supportive; it fundamentally shaped how participants experienced care and navigated uncertainty. Participants also described how even indirect inclusion, such as overhearing respectful professional discussions, created a meaningful sense of involvement for relatives.

These findings suggest that emergency competence in the home is enacted not only technically but relationally, with communication and presence functioning as integral components of clinical safety. Relational attentiveness appears to constitute how safety and emotional steadiness are perceived. This reflects the concept of a caring presence, where professionals' attentiveness contributes to a sense of safety and emotional steadiness [30]. METs' ability to attune to both the patient and the surrounding home context illustrates a form of professional responsiveness that extends beyond routine task performance. Such responsiveness is central to emergency care, especially for vulnerable patients as it situates clinical decision‐making within the lived context of the home. In this way, dignity and connection are embedded in how care is practiced. These findings may be understood in light of philosophies of caring that emphasise presence and attentiveness as foundational to safe and dignified care [30, 31].

In the theme *A sense of safety and security at home*, receiving emergency care at home, rather than in the ED, was associated with emotional calm, dignity and a sense of control, supported by familiar surroundings and the presence of loved ones. This contrast with ED‐based care highlights how the place of care shapes emotional experience, not merely clinical delivery. Previous research has described how the home can function not merely as a physical space but as an active contributor to emotional security and perceived safety [32]. In this study, participants consistently emphasised that supportive and attentive care fostered respect and acknowledgement, suggesting that dignity emerged through interaction within the home context rather than being reducible to location alone.

While home‐based care models are often associated with increased emotional and practical demands on relatives [32], the present study did not indicate that relatives predominantly experienced heightened burden. Instead, their involvement was largely described in positive terms, associated with emotional closeness, clarity and meaningful participation in the care process. This finding nuances existing literature in which caregiver burden is described as a central dimension of family caregiving [33]. Although previous research suggests that strain may increase during care transitions marked by unclear responsibility and limited continuity [34], such heightened burden was not a dominant feature in relatives' accounts. This suggests that perceived strain may be shaped less by location than by how professional responsibility, communication and role boundaries are structured during the encounter. In this way, the study contributes a more differentiated understanding of family involvement in home‐based emergency care.

Although the home setting provided relief and familiarity, the perceived quality of care was not solely determined by location but by how professionalism and presence were enacted by the METs. Participants emphasised that supportive and attentive care fostered a sense of respect and acknowledgement, suggesting that dignity emerged through interaction rather than environment alone. Involving relatives in the conversation further enhanced emotional security, positioning the household as a unit of care. Similar findings have been reported in another care context, where relatives described a need to feel seen, heard and involved during transitions in care [35].

Some participants described uncertainty after the MET had left, highlighting gaps in short‐term interventions without clearly structured follow‐up. This tension between feeling safe during the encounter and uncertain afterward resonates with research describing the vulnerability of clinical handovers, where responsibility involves the exchange of information, accompanied by a transfer of control or responsibility between professionals [36]. Without structured handover processes and coordinated follow‐up, patients may experience fragmentation and insecurity in ongoing care [37]. These findings suggest a structural vulnerability in decentralised emergency models: relational calm achieved during the encounter may not always be sustained without organisational continuity. In the context of home‐based emergency care services, this emphasises the need for integrated and continuous care pathways that extend beyond the immediate intervention to support ongoing safety and coherence.

Taken together, the findings indicate that home‐based emergency care represents more than a simple relocation of services. Rather, it reflects a reconfiguration of emergency practice in which relational presence, contextual sensitivity, and organisational continuity become central to how safety and dignity are perceived and experienced. While relational calm was achieved during the encounter, sustaining that sense of security requires structural continuity. Achieving consistent and sustainable care therefore requires effective communication and coordination across professional roles and organisational levels. Responsibility should extend beyond individual caregivers and be embedded within broader systemic structures that integrate patients' existing care networks [38]. Gaps in structured follow‐up may reflect fragmentation between emergency care and primary or community healthcare services [38]. Without such coordination, the risk of fragmentation remains, potentially undermining the otherwise positive experience during MET visits.

Participants' concerns about post‐MET care, particularly when symptoms persisted, underscore the need for formal structures that bridge acute intervention and ongoing care. This is particularly relevant in healthcare systems increasingly shifting emergency services into community settings, where continuity becomes central to sustaining perceived safety. Without such coordination, the risk of fragmentation remains high.

Realizing the potential of METs therefore depends on ensuring continuity, flexible structures and ethical awareness within home‐based emergency care system [1, 38]. Structured follow‐up models and family participation appear central to sustain calm and dignity beyond the immediate encounter. Supporting professionals with ethical tools and sufficient time for relational care may reinforce professional confidence and strengthen the perceived quality of the care encounter [14]. Both research and organisational development should prioritise improved coordination with primary care and expanded training in communication and ethics, enabling emergency teams to combine clinical competence with compassionate and responsive care.

### 5.1 Strengths and Limitations

A key strength of this study is the inclusion of both patients' and relatives' perspectives, offering a nuanced understanding of emergency care delivered at home. Relatives contributed valuable insights, even though their narratives may not always align fully with those of the patients. The triangulation of perspectives enriched the dataset and strengthened the credibility of the findings [38]. However, involving patients as informants in qualitative interviews poses methodological challenges. Illness, fatigue, or cognitive limitations may influence participants' ability to articulate their experiences in depth [39]. Additionally, the absence of perspectives from MET professionals limits the scope of the findings and raises the question of how their experiences might have complemented the analysis.

The transferability of the findings is shaped by the Swedish context, involving two MET organisations affiliated with hospitals. While this setting enabled a detailed exploration of home‐based emergency care, organisational structures, resources and demographics may vary across regions and countries. For instance, rural areas may face challenges related to distance and access to healthcare services, whereas urban settings may be characterised by high patient flow and shorter interactions. These contextual differences should be considered when applying the findings to other care environments [38]. Nevertheless, as is typical for qualitative research, the goal was not statistical generalisation but the generation of context‐sensitive, transferable insights. The sample size should be considered in relation to the specificity of the study population and the information‐rich data obtained through the interviews [40]. However, a relatively large proportion of eligible individuals declined participation, which may indicate that some of the most vulnerable patients were not represented in the study.

Consequently, the findings may primarily reflect the experiences of individuals who were physically and cognitively able and willing to participate. It cannot be excluded that those who declined participation had different experiences of the care received, including more complex or less positive experiences. Additional participants may therefore have contributed further perspectives and nuances that could have enriched the findings. As a result, some critical perspectives on home‐based emergency care and the care relationship may be underrepresented.

As with all interview‐based studies, the data reflects what participants chose to verbalise, which may not fully capture unspoken dimensions or internal conflicts. In some interviews, patients and relatives participated together, which supported shared recall but may have inhibited the expression of individual or divergent perspectives. Future research could benefit from conducting separate interviews with patients and relatives to further explore potentially differing or conflicting experiences of care. Furthermore, the presence of the interviewer and the relational dynamic during the interviews may have influenced how participants framed their experiences [41]. Two participants were only able to participate in shorter interviews due to illness‐related fatigue. This reflects the vulnerability of the study population and may have limited the depth of individual narratives, particularly among those most affected by illness.

The role of the author group is also preunderstanding warrant's consideration. The first author had prior experience with similar studies and had met some participants during an earlier observational study. While this familiarity may have facilitated rapport and openness, it also carried the risk of shaping both data generation and interpretation. To address this, the first author documented preliminary assumptions and reflections throughout the analytic process. Peer debriefing sessions were conducted at several stages of the analysis, during which developing interpretations were critically discussed and alternative readings were explored. The author group engaged in iterative review of codes and themes, revisiting original transcripts to ensure that interpretations remained grounded in participants' accounts [41].

No formal member checking was conducted. Transcripts were not returned to participants, and they were not invited to provide feedback on the final findings. During interviews, however, participants were encouraged to clarify and elaborate on their perceptions and experiences. The absence of participant validation of the findings may be considered a limitation.

## 6 Conclusions

This study offers new insights into how patients and relatives perceive and experience emergency care at home delivered by METs. The findings demonstrate that safety in home‐based emergency care is not merely a function of clinical expertise but is relationally enacted through communication, presence, and contextual sensitivity. Participants particularly valued METs' calm presence, structured communication, and inclusive approach, which contributed to feelings of safety, involvement, and dignity in a vulnerable situation. The possibility of remaining in a familiar environment further strengthened this sense of security.

Importantly, the study shows that safety in home‐based emergency care depends not only on clinical competence, but also on relational engagement and coordinated continuity beyond the immediate encounter. These findings suggest that emergency care systems must attend to both interpersonal and structural dimensions of care to sustain perceived safety over time.

The findings also clarify the crucial role of relatives in home‐based emergency care. When illness‐related fatigue limited patients' ability to articulate their needs, relatives functioned as advocates and communicative bridges. Recognizing relatives as active partners rather than passive companions is therefore central to delivering safe and responsive emergency care in the home.

At the same time, the findings indicate that home‐based emergency care may involve challenges related to follow‐up and continuity of care. Patients' and relatives' expressed uncertainty regarding where to seek further support after the MET encounter. These aspects highlight the importance of clear communication, written information and coordinated care pathways across healthcare providers and organisations.

Methodologically, the study highlights the challenges of conducting research with frail individuals and underscores the need for adapted data collection approaches. Future research should examine how relational safety can be sustained over time, explore potentially differing experiences between patients and relatives, and investigate organisational barriers and follow‐up structures needed to support coherent and integrated care pathways.

## Author Contributions

Å.F., G.N.B., H.A. and A.S. developed the study design. Å.F. conducted the interviews. Å.F. performed the data analysis and interpretation, which were further discussed with H.A. and U.A. Å.F. drafted the manuscript, with A.S., G.N.B., H.A. and U.A. providing ongoing supervision. All authors read and approved the final manuscript.

## Funding

The authors have nothing to report.

## Ethics Statement

Ethical approval was obtained from the Swedish Ethical Review Authority in Stockholm (No: 2023‐02186‐01), and from relevant clinical operations managers.

## Consent

All participants provided informed consent prior to data collection.

## Conflicts of Interest

The authors declare no conflicts of interest.

## Data Availability

The data that support the findings of this study are available from the corresponding author upon request.
